# Sleep of Medically Ill Hospitalized Older Adults: Do Sleep Medications Make a Difference?

**DOI:** 10.1093/geroni/igaf122.1917

**Published:** 2025-12-31

**Authors:** Juliana Smichenko, Tamar Shochat, Anna Zisberg

**Affiliations:** University of Haifa, Haifa, HaZafon, Israel; University of Haifa, Haifa, HaZafon, Israel; University of Haifa, Haifa, HaZafon, Israel

## Abstract

Sleep disturbances are common among medically ill older adults during acute hospitalization, often leading to adverse outcomes. Despite frequent use of sleep medications, their effectiveness remains uncertain. This study examined changes in sleep parameters from home to hospital and evaluated the impact of physical symptom burden and sleep medications burden daily trajectories as key factors. A prospective multicenter study was conducted across four Israeli hospitals. The study included 683 cognitively intact older adults who completed an admission interview and at least one follow-up. Sleep parameters—including total sleep time (TST), sleep efficiency (SE), sleep quality (SQ), and number of awakenings (NOA)—were assessed daily, along with sleep medication use and burden (dosage and quantity). Physical symptom burden and additional covariates were analyzed using a repeated measures mixed models approach. Participants (54% male, mean age 77.31±6.60) experienced reduced TST (329.73±111.94 vs. 377.03±101.06 minutes), lower SE (71.49±19.28% vs. 76.14±15.53%), and poorer SQ in the hospital compared to home. Sleep medication use was not associated with any sleep parameter, while sleep medication burden correlated with increased NOA. Physical symptom burden had significant effects on SE, SQ, and NOA. A time-dependent interaction showed that higher symptom burden was most strongly linked to reduced TST at the first in-hospital follow-up, stabilizing thereafter. Sleep deteriorates during hospitalization, with minimal benefit from sleep medications, however stronger effect of physical symptoms severity. Prioritizing symptom management may be key to improving sleep in hospitalized older adults.

